# Bibliometric Analysis of Islamic Crowdfunding: A Literature Review of Its Journey

**DOI:** 10.12688/f1000research.146797.1

**Published:** 2024-05-24

**Authors:** Aris Purwatiningsih, S Purnamasari, Harini Setyawati, Astiwi Indriani, Dian Prawitasari, Shoimatul Fitria

**Affiliations:** 1 Economic, Universitas Dian Nuswantoro, Semarang, Central Java, 50131, Indonesia; 2 Islamic Economy, Universitas Islam Kalimantan Muhammad Arsyad Al Banjari, Banjarmasin, South Kalimantan, 70123, Indonesia; 3 Economic, Universitas Putra Bangsa, Kebumen, Jawa Tengah, 54361, Indonesia; 4 Economic, Universitas Diponegoro, Semarang, Central Java, 50275, Indonesia

**Keywords:** Bibliometric analysis, Islamic crowdfunding, Legislation

## Abstract

**Background:**

The potential of Islamic crowdfunding to encourage the development of Islamic fintech globally, even in countries with non-Muslim majority populations, needs to be examined in a literature study on this issue. More extensive research is needed regarding the factors that most reliably predict the success of Islamic crowdfunding, such as compliance with Islamic crowdfunding laws, sustainability, and the potential of Islamic finance. This article describes a comprehensive and systematic Literature Review (SLR) regarding papers published in the field of Islamic crowdfunding. This research aims to contribute to a better understanding of Islamic crowdfunding, provide useful information for practitioners, and stimulate further research in the field to increase the success of Islamic crowdfunding.

**Methods:**

We conducted a review of selected papers to identify gaps and significant issues in Islamic crowdfunding, providing guidance for future researchers. This article review was based on 704 articles retrieved using the keyword “Islamic crowdfunding” from the Scopus database between 2013 and 2022. To minimize bias, we formulated 5 research questions to guide our analysis: RQ1: How do publication and citation rates vary annually? RQ2: How can we determine the most influential studies based on citation counts for each keyword? RQ3: What are the most common subject areas addressed in Islamic crowdfunding literature from 2013 to 2022? RQ4: What are the most frequently used keywords in Scopus documents? RQ5: How are Islamic values discussed in articles related to Islamic crowdfunding?

**Results:**

The findings of this research demonstrate that Islamic crowdfunding, characterized by interdisciplinary scholarship, has emerged as an increasingly significant alternative for aiding society and the economy. It contributes to poverty alleviation and the development of specific sectors through channels such as crowdfunding for small and medium enterprises and social projects tailored to meet the diverse needs of Muslims. However, the challenge facing the future development of Sharia crowdfunding is that, despite its benefit of increasing community participation in projects aligned with religious values, it also carries the potential risk of investment losses and non-compliance with Sharia principles. Ways to address the current scarcity of Sharia fintech are also needed, ensuring its broader acceptance across all societal levels, and enhancing Muslims’ understanding of compliance with Islamic religious rules, particularly Fiqh law.

**Conclusion:**

Urgently needed are specific fiqh regulatory guidelines to ensure that Sharia-compliant crowdfunding adheres to Islamic principles. Government support, particularly through legislation in countries where the majority of the population is Muslim, is crucial to enhance public participation and trust in Sharia crowdfunding.

## Introduction

Islamic crowdfunding is a form of crowdfunding that operates in accordance with Islamic principles and ethics. It integrates the features of a crowdfunding platform with Islamic crowdfunding and business regulations to facilitate the funding of projects and initiatives aligned with Islamic values. Islamic crowdfunding platforms utilize blockchain technology to ensure transparency and traceability of transactions ( Kuanova, Sagiyeva, and Shirazi, 2021). These platforms provide a digital space where individuals can donate or invest in projects promoting social welfare and entrepreneurship ( Hendratmi, Ryandono, and Sukmaningrum, 2020), education, or other areas of interest within the Islamic community. Consequently, Islamic crowdfunding has the potential to support various sectors, including book publishing ( Ishak and Rahman, 2021), contribute to sustainable development ( Testa *et al*., 2019), affordable housing ( Ma and Md Taib, 2023), and zakat donations ( Adnan *et al*., 2023).

By leveraging technology and Islamic finance principles, Islamic crowdfunding provides a unique avenue for individuals to participate in initiatives that are both socially responsible and financially inclusive ( Testa *et al*., 2022). Several challenges may impede the development and progress of Islamic crowdfunding, including Sharia supervision ( Dawood *et al*., 2022), a lack of awareness and understanding of the concept and potential of Sharia crowdfunding among the public ( Vaznyte, Andries, and Manigart, 2023), the absence of clear regulations and internationally recognized standards ( Hendratmi, Ryandono, and Sukmaningrum, 2020), a lack of strong relationships with potential donors and effective electronic word-of-mouth marketing ( Alalwan *et al*., 2017). Furthermore, there is a need for the implementation and understanding of the use of Islamic crowdfunding in managing Islamic funds and the limited number of registered Islamic crowdfunding platforms ( Ag Omar *et al*., 2021). Additionally, there is a need for a more Sharia-compliant platform and an emphasis on financing Sharia in Sharia crowdfunding ( Ramli, Abdullah, and Alam, 2023), as well as consistency and transparency in Sharia compliance ( Ishak and Rahman, 2021).

Islamic crowdfunding is a relatively new business concept, even in Muslim-majority countries, and there is still limited literature on Islamic fintech ( Shaikh *et al*., 2020). For example, Malaysia lacks a comprehensive understanding of its society, and the studies on Islamic crowdfunding and qualitative analyses are limited ( Ishak and Rahman, 2021). Additionally, there are constraints related to the number of sources and the scope of studies conducted on Islamic crowdfunding ( Ishak, Kamaruddin, and Aderemi, 2022), hindering the optimal success of Islamic crowdfunding. Challenges such as a lack of awareness and understanding of Islamic crowdfunding, insufficient studies on the subject, and the early stages of development in regulatory and legal frameworks for Sharia-compliant Islamic crowdfunding also contribute to the challenges facing the success of Islamic crowdfunding in Indonesia, a country with the largest Muslim population globally ( Darmansyah *et al*., 2020). Further research and exploration are imperative to fully comprehend and leverage the potential of Islamic crowdfunding. Hence, this literature review on Islamic crowdfunding aims to enhance understanding, provide valuable information for practitioners, and encourage additional research in the field to support the development of a more effective crowdfunding model in line with Sharia principles, ultimately boosting the success of Islamic crowdfunding in the broader community.

The absence of studies on Islamic crowdfunding, limited awareness among publications, and the absence of a Sharia-compliant regulatory and legal framework pose challenges to the success of Islamic crowdfunding in Indonesia, a nation with the largest Muslim population globally, and these challenges are still in their early stages of development. Islamic crowdfunding has the potential to positively impact various industries, including publishing, community-based tourism, and microbusinesses. To fully grasp and harness the potential of Islamic crowdfunding, more investigation and study are required. Therefore, the purpose of this literature review on Islamic crowdfunding is to stimulate further research in the field, furnish practitioners with valuable information, and foster a better understanding of the subject. This will contribute to the creation of a more effective crowdfunding model that adheres to Sharia principles, ultimately increasing the success of Islamic crowdfunding in the broader community.

This paper aims to provide a better understanding of Islamic crowdfunding, offer valuable information for practitioners, and promote further research in this field to support the development of improved crowdfunding models in accordance with Sharia principles to enhance the success of Islamic crowdfunding in Indonesia. To achieve this goal, we address the following Research Questions:

RQ1. How do publications and citations perform annually?

RQ2. How can we identify the most influential studies based on the number of citations for each keyword?

RQ3. What are the most common subject areas found in Islamic crowdfunding from 2013 to 2022?

RQ4. What are the most used keywords in Scopus documents?

RQ5. How are Islamic values discussed in Islamic crowdfunding articles?

## Background

The development of Islamic finance can be harnessed to support Islamic social financial instruments through technological applications. This enables efficient and effective administration, collection, and distribution of Islamic social funds ( Umar *et al*., 2022), thereby supporting the success of ziswaf ( Ascarya and Sakti, 2022), and SUKUK ( Chong, 2021). It is essential to segregate ZISWAF funds from funds of other fintech users to prevent the mingling of funds with other funding sources ( Indahsari *et al*., 2014). The integration of technology with zakat represents a crucial social financial tool for alleviating global poverty, exemplified by the adoption of ZakaTech by Muslims during COVID-19 ( Abdullah *et al*., 2023).

Islamic crowdfunding, operating in accordance with Islamic principles and ethics, combines crowdfunding platforms with Islamic crowdfunding and business rules to facilitate financing for projects aligned with Islamic values. These platforms employ blockchain technology to ensure transparency and traceability of transactions ( Alshater *et al*., 2022), providing a digital space where individuals can donate or invest in projects promoting social welfare, entrepreneurship, education, or other areas of interest. In this context, Islamic crowdfunding has the potential to support various sectors, including book publishing ( Ishak and Solihin, 2012), contribute to sustainable development ( Testa *et al*., 2019), affordable housing, and zakat donations ( Beik and Arsyianti, 2016). By leveraging technology and Islamic finance principles, Islamic crowdfunding offers a unique pathway for individuals to participate in socially responsible and financially inclusive initiatives. It is a relatively new business, even in Muslim-majority countries, where literature on Islamic fintech is still limited ( Shaikh *et al*., 2020). Malaysia, for example, lacks a comprehensive understanding of Malaysian society, empirical studies on Islamic crowdfunding, and qualitative analysis, as well as faces limitations in the number of sources and scope of studies on Islamic crowdfunding ( Ramli, Abdullah, and Alam, 2023), resulting in suboptimal success for Sharia crowdfunding.

The challenges to the success of Sharia crowdfunding in Indonesia, despite being the country with the largest Muslim population globally, include a lack of awareness and understanding of Sharia crowdfunding among publications, insufficient studies on Sharia crowdfunding, and an early-stage regulatory and legal framework for Sharia crowdfunding ( Darmansyah *et al*., 2020). Several challenges hindering the development and progress of Sharia crowdfunding include a lack of awareness and understanding of the concept and potential of Sharia crowdfunding among publications, unclear regulations and internationally recognized standards, weak relationships with potential donors, and underutilization of electronic word of mouth. There is also a lack of implementation and understanding of Sharia crowdfunding in managing Sharia funds and a limited number of registered Sharia crowdfunding platforms ( Alsmadi *et al.*, 2023).

Apart from the aforementioned problems, the current significant challenge lies in building strong relationships with potential donors and leveraging the influence of electronic word of mouth (eWOM) ( Torres, Augusto, and Wallace, 2018). There is also a need for more Sharia-compliant platforms and an emphasis on Sharia financing in Sharia crowdfunding, along with consistency and transparency in Sharia compliance, publishing, zakat, and qardul hasan ( Ali, 2017). Further research and exploration are necessary to understand and exploit the potential of Islamic crowdfunding. Therefore, this literature review on Islamic crowdfunding aims to provide a better understanding of the topic, furnish valuable information for practitioners, and encourage further research in this area to support the development of improved crowdfunding models in accordance with Sharia principles, ultimately increasing the success of Sharia crowdfunding in the wider community.

### Crowdfunding and Islamic finance

Islamic crowdfunding campaigns are crucial for the hotel industry, which has been adversely affected by the COVID-19 pandemic ( Baber *et al*., 2022). There is a need for better alternatives to enhance crowdfunding trust among donors for strategic sustainability in the conditions of turbulence and financial crisis caused by COVID ( Jiao *et al*., 2022). Crowdfunding is a financial phenomenon aimed at obtaining resources to implement innovative projects in a market where success is determined by experience, human capital, and gender ( Borrero-Domínguez, Cordón-Lagares, and Hernández-Garrido, 2020). Performance and effort expectations, social influences, as well as facilitating conditions significantly influence entrepreneurs’ behavioral intentions to adopt crowdfunding, although its reception may not be as favorable as in developed countries due to the lack of supporting tools, financial regulations, and promotional campaigns ( Islam and Khan, 2021). Crowdfunding platforms can also contribute to sustainable development processes ( Testa *et al*., 2022) by supporting green projects, environmental protection, and sustainability initiatives ( Prędkiewicz and Kalinowska-Beszczyńska, 2021).

In the initial stages of creating a crowdfunding platform, social capital is required to support the success of the campaign ( Butticè, Colombo, and Wright, 2017). Equity crowdfunding is a viable opportunity to finance new businesses, especially startups and small and medium enterprises (SMEs) ( Giudici, Guerini, and Rossi-Lamastra, 2020). Crowdfunding has the potential to democratize access to finance and provide new investment opportunities, including for underrepresented groups such as women ( Serwaah, 2022). The success of previous campaign programs affects individual empathy and intention to donate to crowdfunding charities ( Lin *et al*., 2018). Islamic crowdfunding platforms (Islamic crowdfunding FPs) have the potential to contribute to sustainable development by embedding social responsibility and providing fundraising opportunities for unbanked and underserved individuals ( Testa *et al*., 2022). Islamic crowdfunding is part of the global Islamic crowdfunding economy, thereby providing opportunities for crowdfunding initiatives ( Floren, Rasul, and Gani, 2020). Sharia crowdfunding strategies are a solution to economic and social welfare problems in various countries. This strategy leverages the use of crowdfunding platforms and blockchain technology to ensure transparency and track transactions, in line with the ethical norms and rules of Islamic crowdfunding business ( Kuanova, Sagiyeva, and Shirazi, 2021).

### Fintech and blockchain

The utilization of fintech as a microfinance initiative offered by Islamic banks needs to be increased to promote ṣadaqah-based loans and products ( Abd Wahab *et al*., 2023). Fintech can be a solution and support the resilience of the sharia crowdfunding banking sector in addressing the challenges faced in the context of the COVID-19 pandemic ( Pireh, 2022). The government and society are encouraged to strengthen and expand social networks, particularly to enhance crisis management for recovery from the impact of the COVID-19 pandemic ( Pitas and Ehmer, 2020). Similar to the innovations implemented by companies in Brunei Darussalam to maintain the resilience and continuity of their businesses during the pandemic ( Basir, 2022), sharia financial instruments such as waqf play a role in providing health equipment and assisting those affected by the pandemic ( Kasri and Chaerunnisa, 2021).

The use of ZakaTech has the potential to enhance the fundraising capacity of Islamic crowdfunding social financial institutions ( Bin-Nashwan *et al*., 2022). The impact of fintech developments has also manifested in Islamic finance and economics, positioning Islamic crowdfunding as a provider of various solutions to the economic impact of COVID-19 through key principles and investment tools, including profit-sharing, takaful, and waqf ( Benamraoui, 2021). Factors driving the adoption of fintech services, such as perceptions of trust, exert a highly significant direct influence on the intention to use Islamic Paytech crowdfunding services ( Irimia-Diéguez *et al*., 2023). Other driving factors include perceived ease of use, perceived usefulness, and consumer innovation ( Shaikh *et al*., 2020). The presence of crowdfunding has a positive impact on the success of fundraising, and crowdfunding platforms are effective fintech tools for financing entrepreneurs in the Middle East ( Abdeldayem and Aldulaimi, 2022).

Islamic fintech provides opportunities for new start-ups with increased transparency and ethical practices in Islamic crowdfunding financial institutions, thereby enhancing processes and providing cost-effectiveness in delivering sharia-compliant products and services ( Chong, 2021). Islamic crowdfunding platforms, combined with blockchain technology, can promote transparency and facilitate the growth of Islamic crowdfunding social instruments and projects, involving stakeholders such as governments, Islamic crowdfunding financial institutions, and commercial organizations ( Kuanova, Sagiyeva, and Shirazi, 2021). In Islamic crowdfunding with blockchain technology, the occurrence of mudharabah financing based on crowdfunding and murabahah is highly possible ( Ag Omar *et al*., 2021).

### Islamic crowdfunding

Islamic crowdfunding is a form of crowdfunding that operates in accordance with Islamic principles and ethics. It combines the use of crowdfunding platforms with Islamic contracts and business rules to facilitate the financing of projects and initiatives that align with Islamic values. Islamic crowdfunding platforms leverage blockchain technology to ensure transparency and traceability of transactions ( Kuanova, Sagiyeva, and Shirazi, 2021), providing a digital space where individuals can donate or invest in projects promoting social welfare, entrepreneurship, education, or other areas of interest, including in the book publishing sector ( Ag Omar *et al*., 2021).

Islamic crowdfunding platforms (Islamic crowdfunding FPs) contribute to sustainable development ( Testa *et al*., 2022), affordable housing ( Ma and Md Taib, 2023), zakat donations waqf ( Hapsari *et al*. 2022), and qordul hasan ( Pireh, 2022). By leveraging technology and Islamic finance principles, Islamic crowdfunding offers a unique pathway for individuals to participate in socially responsible and financially inclusive initiatives ( Testa *et al*., 2022). Additionally, the use of fintech platforms and technology has become increasingly prominent during the pandemic, including in the field of Islamic finance ( Darmansyah *et al*., 2020).

Sharia crowdfunding can optimize the role of sharia fintech, such as the crowdfunding model, as an intermediary between Muslim donors or crowd funders and micro, small, and medium enterprises (MSMEs) ( Raimi and Uzodinma, 2020). Overall, Islamic crowdfunding strategies provide an innovative approach to overcome economic problems and improve social welfare in various countries. The acceptance and success of Islamic crowdfunding are influenced by factors such as social capital, religiosity, social influence, and demographic characteristics of Islamic crowdfunding ( Choy and Schlagwein, 2016). Islamic crowdfunding can also be used for various purposes, including affordable housing solutions, entrepreneurial development, and investment opportunities, offering an alternative model for Islamic crowdfunding venture capital companies by providing a mechanism to mitigate business risks ( Gafrej and Boujelbéne, 2022).

The multidimensional model of high-growth companies in Russian-Ukrainian Islamic crowdfunding consists of several interrelated dimensions, such as innovation, finance, human resources, and business strategies after the COVID-19 pandemic, which are very different from the previous period ( Tchinda and Dejardin, 2021). Overall, Islamic crowdfunding is used globally for a variety of purposes, namely meeting economic, social, and charitable needs. Several challenges that can hinder the development and progress of sharia crowdfunding are: lack of awareness and understanding of the concept and potential of sharia crowdfunding among publications ( Alalwan *et al*., 2017); the absence of clear regulations and internationally recognized standards ( Darmansyah *et al*., 2020); lack of strong relationships with potential donors and harnessing the electronic power of word of mouth ( Alalwan *et al*., 2022); implementation and understanding of the use of sharia crowdfunding in managing sharia funds and the limited number of registered sharia crowdfunding platforms; as well as the need for more sharia-compliant platforms and the importance of Islamic crowdfunding ( Beck, Demirg-Kunt, and Merrouche, 2013); also consistency and transparency in Sharia compliance ( Ramli, Abdullah, and Alam, 2023).

Islamic crowdfunding platforms contribute to the growth of Islamic crowdfunding instruments and social projects, overcoming economic challenges and improving social welfare ( Herianingrum *et al*., 2022). Islamic crowdfunding and blockchain technology are becoming mainstream in the Muslim world, especially for small and medium enterprises (SMEs) ( Hudaefi, 2020). Islamic crowdfunding has the potential to manage Islamic funds, such as cash waqf and zakat, effectively ( Indahsari *et al*., 2014). Islamic crowdfunding platforms, such as EthisCrowd, provide affordable housing solutions for low-income households in Indonesia ( Ma and Md Taib, 2023). Knowledge, belief, and religiosity play a positive role in explaining the intention to donate money online among the Indonesian millennial generation ( Kasri and Chaerunnisa, 2021). Crowdfunding campaigns that combine religious and social narratives are more likely to attract funding and achieve success ( Simpson *et al*., 2020).

Several factors determine the success of Islamic crowdfunding, including perceptions of other people’s charitable behavior, previous donations, religiosity, knowledge, beliefs, and attitudes towards the intention to donate to cash waqf—a reflection of strong Islamic values that prioritize religion, increasing literacy waqf, trust in cash waqf institutions, and developing effective marketing strategies. Knowledge and effective communication of religious messages, beliefs, and religiosity play a positive role in explaining the intention to donate money online among the Indonesian millennial generation ( Kasri and Chaerunnisa, 2021).

## Methods

This research collects data from the Scopus database, which contains more Islamic finance research papers and indexes more validated research data ( Alshater *et al.*, 2021). We reviewed and re-checked the database to verify the suitability of the data for processing. Additionally, we re-read and analyzed the data according to predetermined data categorization. At last, we conducted a subgroup analysis for identify if there were any differences in results between the predetermined groups, then We assessed whether certain characteristics, such as study characteristics or population characteristics, significantly influenced the results. Furthermore, we conducted a review based on 704 articles retrieved using the keyword “Islamic crowdfunding” from the Scopus database between 2013 and 2022. To minimize bias, we formulated 5 research questions to guide our analysis: RQ1: How do publication and citation rates vary annually? RQ2: How can we determine the most influential studies based on citation counts for each keyword? RQ3: What are the most common subject areas addressed in Islamic crowdfunding literature from 2013 to 2022? RQ4: What are the most frequently used keywords in Scopus documents? RQ5: How are Islamic values discussed in articles related to Islamic crowdfunding?

## Results

The majority of the publications fall under the category of “ Article,” constituting 67.33% of the total. This suggests a significant focus on standard articles, which are typically in-depth research papers. Book Chapter make up a smaller proportion at 17.77%. Table 1 also suggest that a substantial involvement in contributing to books or edited volumes, which often include specialized topics. Conference papers make up a smaller proportion at 6.53%. This indicates a presence of research contributions presented at conferences, which may have a more condensed format compared to full articles. The “Book” category represents 5.68%, suggesting an involvement in writing entire books. This can be a significant contribution, indicating a comprehensive exploration of a particular subject. Reviews constitute 3.98%, indicating that there is some engagement in critically evaluating and summarizing existing literature in the field. Editorials make up a minimal percentage at 0.06%. Highlighting a limited but detectable representation in the dataset. Editorials are often opinion pieces or introductory notes written by the editorial team of a publication. Short survey, Note, and Conference Review have the smallest representation at 0.01%. This suggests a minimal focus on Short survey, Note, and Conference Review publications, which are usually brief overviews of a specific topic.

**Table 1.** Document type.

Document type	Total publications (TP)	Percentage (%)
Article	473	67.33
Conference Paper	46	6.53
Book Chapter	111	17.77
Note	1	1.42
Review	28	3.98
Editorial	4	0.06
Book	40	5.68
Short Survey	1	0.01
**Total**	**704**	**100.00**

In summary, the majority of publications consist of standard articles and book chapters, indicating a strong emphasis on in-depth research. Conference papers, books, and reviews also contribute, showcasing a diverse range of scholarly outputs. There are also editorials, short surveys, notes, and conference reviews, although their numbers are very limited. The data reflects a balanced approach to academic publishing, covering various formats and contributing to different types of publications.

The majority of publications come from journals, constituting 57.30%. This suggests a significant focus on disseminating research through peer-reviewed journals, which is a common and authoritative way to share academic work. Books and book series make up a substantial portion at 5.95% and 7.30%, indicating an involvement in writing standalone books and book series. This may include monographs or comprehensive works on specific topics. Publications in Conference Proceeding contribute 28.11%. This indicates active participation and contribution to academic conferences, where research is presented and published in proceedings.

In summary, the data in Table 2 shows a diverse range of source types, with a primary focus on journals and books. The distribution suggests a balanced approach to publishing, involving both traditional academic outlets (journals and books) and other forms of scholarly communication book series, and books).

**Table 2.** Source type.

Source type	Total publications (TP)	Percentage (%)
Journals	212	57.30
Conference Proceedings	104	28.11
Book Series	27	7.30
Books	22	5.95
Trade Publications	5	1.35
**Total**	**370**	**100.00**

The overwhelming majority of publications are in English, constituting 97.30%. This is consistent with the global dominance of English as the primary language for scholarly communication. German (1.08) are the second most represented language. This suggests a smaller but notable presence in publications in the German language. Other languages such as Chinese French, and Spanish, each (0.27), indicate that there are still articles written in these languages, although the number is very small.

In summary, the data in Table 3 shows a strong emphasis on English-language publications, reflecting the international and widespread nature of academic research. The presence of publications in other languages, albeit at a smaller percentage, suggests a degree of diversity in language representation, possibly due to specific regional or disciplinary focuses.

**Table 3.** Languages.

Language	Total publications (TP)	Percentage (%)
English	361	97.30
German	4	1.08
Portuguese	3	0.81
Chinese	1	0.27
French	1	0.27
Spanish	1	0.27
**Total**	**371**	**100.00**

Subject Area from Table 4 suggests a strong focus on Business, Management, and Accounting, along with significant contributions to Economics, Econometrics and Finance Social Sciences, Computer Science, Decision Science, and other areas.

1. Business, Management and Accounting (30.04%): This subject area has the highest percentage of publications, indicating a significant focus on topics related to business, management, and accounting.
2. Economics, Econometrics and Finance (25.81%): Another substantial area, suggesting a strong presence in economic and financial research.
3. Social Sciences (13.40%): Reflects a notable contribution to the social sciences.
4. Computer Science (12.13%): Indicates a significant involvement in computer science research.
5. Decision Science (8.95): indicates that decision science a noteworthy representation.
6. Energy (7.24): indicates that energy reflects a quite prominent presence in the field of study.
7. Environment Science (6.82): Informs Environment Science is still often a subject of discussion.
1. Engineering (5.92%): Represents a presence in engineering research.
2. Mathematics (3.55): Suggesting the involvement of mathematics in critically evaluating and summarizing existing literature.
3. Arts and Humanities (3.24%): Indicates a smaller but existing contribution to arts and humanities.
4. Psychology (1.99), Medicine (1.56), Physics and Astronomy (1.42), Multidisciplinary and Agricultural and Biological Sciences each (1.14): Represent various fields with moderate contributions.
5. Other Areas (Earth and Biological Science, Materials Science, Biochemistry, Genetics, and Molecular Biology, Pharmacology, Toxicology and Pharmaceutics, Immunology and Microbiology, Health Professions, Chemical Engineering, Veterinary, Neuroscience): Reflects diverse interests with smaller percentages.

**Table 4.** Subject area.

Subject area	Total publications (TP)	Percentage (%)
Business, Management and Accounting	361	30.04
Economics, Econometrics and Finance	286	25.81
Social Sciences	226	13.40
Computer Science	131	12.13
Engineering	71	5.92
Engineering	71	5.36
Arts and Humanities	23	3.24
Decision Science	63	8.95
Energy	51	7.24
Environmental Science	48	6.82
Mathematics	25	3.55
Psychology	14	1.99
Medicine	11	1.56
Physics and Astronomy	10	1.42
Multidisciplinary	8	1.14
Agricultural and Biological Sciences	8	1.14
Earth and Biological Sciences	6	0.85
Materials Science	5	0.71
Biochemistry, Genetics, and Molecular Biology	4	0.57
Pharmacology, Toxicology and Pharmaceutics	2	0.28
Immunology and Microbiology	2	0.28
Health Professions	2	0.28
Chemical Enginering	2	0.28
Veterinary	1	0.14
Neuroscience	1	0.14

Keywords Based on Table 5, indicates specific areas of interest, with a substantial emphasis on crowdfunding, fintech, and blockchain-related research.

1. Crowdfunding (67.33%): Indicates a dominant focus on crowdfunding-related topics
2. Fintech (6.53%): Represents a notable presence in financial technology research.
3. Blockchain (17.77%): Suggests a significant focus on blockchain-related topics.
4. Malaysia (1.42%), Review (3.98), Book (5.68): Indicates a smaller but existing focus on research related to Islamic Crowdfunding.
5. Other Keywords (Editorial and Short Survey): Contribute to the diversity of topics covered, with various percentages.

**Table 5.** Filter by keyword.

Keyword	Total Publications (TP)	Percentage (%)
Crowdfunding	74	67.33
Fintech	53	6.53
Blockchain	39	17.77
Malaysia	26	1.42
Review	28	3.98
Editorial	4	0.06
Book	40	5.68
Short Survey	1	0.01
**Total**	**704**	**100.00**

## Discussion

### Publication and citations perform annually

We began analyzing documents with the keyword “Islamic crowdfunding” in 2013 because the crowdfunding model had successfully transformed the way society funds projects, businesses, and social initiatives. This shift captured the attention of academics seeking to comprehend the impact and potential of this innovative financing model. Our database search commenced with the keyword “Islamic crowdfunding” in 2013 because we discovered 1 document on Islamic crowdfunding in the Scopus database for that year, which had not been found in previous years.

Sharia finance is a financial sector adhering to Islamic sharia principles. Islamic finance had been rapidly developing before 2013, and academics were intrigued by the prospect of integrating sharia concepts with the emerging crowdfunding trend. This integration aimed to create a financing model aligned with Islamic principles. There was a surge in awareness and interest in financial products complying with Islamic principles, prompting Muslim communities worldwide to explore investment or project funding options in accordance with their beliefs. Islamic crowdfunding initiatives emerged as an interesting alternative in this context.

The potential of financial inclusion to provide access to individuals or groups that previously faced difficulties or lacked access to formal financial markets intrigued academics. They sought to explore how crowdfunding models, including sharia-based ones, could enhance financial well-being and inclusion in Muslim communities. This research not only facilitates access for individuals or groups that previously encountered financial difficulties or lacked access to formal financial markets but also sparks academic interest in investigating further the realm of Islamic crowdfunding. It underscores an increase in interest, utilization, and growth of crowdfunding as a financing method. However, it is crucial to note the limitations of crowdfunding in educational campaigns during this period.

Post-2013, there was a growing body of research on this subject, with a notable surge during the COVID-19 pandemic in 2020. This surge was prompted by shifts in spending patterns and heightened humanistic values in society during the pandemic. The phenomenon underscored the need for mutual assistance, resulting in a substantial amount of aid being provided. These events motivated researchers to delve into this phenomenon, evident in the rising number of documents in the database from 2013 to 2022. The progression was particularly remarkable from 2016 to 2022, with the number of documents increasing from 8 to 295. This surge in documents in the Scopus database was accompanied by a corresponding increase in citations, notably peaking from 2019 to 2022.

### Identify the most influential studies

There are six most common fields of study in Scopus documents, namely: Business, Management and Accounting, Economics, Econometrics and Finance, Social Sciences, and Computer Science. Research on Islamic crowdfunding focuses significantly on Business, Management, and Accounting for several reasons. First, Islamic crowdfunding is a financial activity that requires expertise in financial management, risk management, and marketing strategies, as seen in Islamic banks ( Aderemi and Ishak, 2020). Managing funds and resources is crucial in crowdfunding, including Islamic crowdfunding. Additionally, knowledge in financial management and accounting practices is essential for Islamic crowdfunding ( Hasan, Hassan, and Rashid, 2018). Islamic crowdfunding studies also intersect with economics and business management, involving the analysis of banking effectiveness, financial literacy, and customer awareness ( Albaity and Rahman, 2019). The success of a crowdfunding campaign, especially in the environmentally friendly project sector, depends on factors like campaign characteristics, updates, and the targeted amount. Islamic crowdfunding requires an understanding and application of marketing and management principles ( Prędkiewicz and Kalinowska-Beszczyńska, 2021). Therefore, the involvement of business and management disciplines in Islamic crowdfunding research is essential to comprehend its dynamics and optimize outcomes.

Research on Islamic crowdfunding also places a significant focus on Economics, Econometrics, and Finance for several reasons. Firstly, crowdfunding plays a crucial role in economic development, especially in the Gulf Cooperation Council (GCC) countries ( Thottoli, 2022). Secondly, crowdfunding adoption among start-up entrepreneurs, particularly in developing countries, is still low, necessitating research to identify influencing factors such as performance expectations, effort expectations, social influence, facilitating conditions, and perceived trust ( Islam and Khan, 2021). Furthermore, crowdfunding is not solely an economic phenomenon but also involves social relationships and motivation ( Sherman and Axelrad, 2022). Aspects like social trust, networks of friends and family, and social enterprise are integral to crowdfunding ( Flanigan, 2017). Additionally, crowdfunding has emerged as an additional source of financing for research projects. Therefore, understanding the factors influencing the success of scientific crowdfunding projects, including newsworthiness, reputation signalling, and online payment theory, is crucial ( Schäfer *et al*., 2016). Overall, research on Islamic crowdfunding encompasses various economic and financial aspects to understand its role, adoption, and impact.

Research on Islamic crowdfunding also involves a significant focus on Social Sciences for several reasons. Firstly, Islamic crowdfunding often supports creative projects in fields such as literature, art, and media ( Alblooshi, 2022). For example, a crowdfunding platform was used to fund the publication of Ishak *et al*.’s book ( Aderemi and Ishak, 2020) and independent print media ( Konhäusner *et al*., 2021). These projects rely on the support and contributions of individuals who value and appreciate the arts and creative endeavours. Crowdfunding campaigns often involve storytelling and community engagement, which are important elements of the arts and humanities. Crowdfunding platforms provide opportunities for creators to connect with their audiences, build communities, and share their vision and passion. Research on crowdfunding explores the motivations and behavior of backers and contributors ( Steigenberger, 2017). To understand the human aspects of crowdfunding, such as the reasons why people contribute and the impact of crowdfunding on society, requires a focus on the arts and humanities. By studying these aspects, researchers can gain insight into the social and cultural dynamics of Islamic crowdfunding and its impact on creative industries and communities.

Computer Science is a field related to the development of Islamic crowdfunding because crowdfunding platforms heavily rely on website platforms and web-based online platforms ( Jiao *et al*., 2022). Computer science also plays a crucial role in ensuring transparency and transaction tracking in crowdfunding platforms, especially when combined with blockchain technology. Computer science is essential for developing and analyzing data in the context of crowdfunding, allowing researchers to understand behavioral intentions and factors influencing the adoption of Islamic crowdfunding services ( Alsmadi, Alrawashdeh, *et al.*, 2023). Overall, computer science is an integral part of the functioning, development, and analysis of Islamic crowdfunding platforms. Relationship marketing orientation, entrepreneurial alertness, system quality, and service quality significantly influence entrepreneurs’ involvement in e-equity crowdfunding, predicting knowledge acquisition and innovation performance ( Pratono *et al*., n.d.). Computer science and science can address challenges in the fields of computers, science, and technology but have not been able to improve a person’s ethics ( Oliver and McNeil, 2021). All in all, computer science is an integral part of the functioning, development, and analysis of Islamic crowdfunding platforms.

### The most common subject areas found in Islamic crowdfunding in 2013-2022, will these three subjects always be present in every study?

Crowdfunding plays a major role in economics development, particularly in the Gulf Cooperation Council (GCC) countries serves as a source of financing for new business startups and small and medium enterprises (SMEs). The adoption of crowdfunding among startup entrepreneurs, especially in developing countries, is still low. Therefore, the research aims to identify the factors that influence crowdfunding adoption, such as performance expectations, effort expectations, social influence, facilitating conditions, and perceived trust. In addition, crowdfunding is not solely an economics phenomenon but involves social relations and motivation ( Sherman and Axelrad, 2022). Social trust, network of friends and family, and social ventures are important aspects of crowdfunding ( Flanigan, 2017). In addition, crowdfunding has emerged as an additional source of financing for research projects. It is important to understand the factors that influence the success of scientific Islamic crowdfunding projects, including news value, reputation signalling and online payment theory ( Schäfer *et al*., 2016). Overall, research on Islamic crowdfunding covers various economics and financial aspects to understand its role, adoption, and impact.

Research on Islamic crowdfunding involves many Social Sciences, for several reasons. First, Islamic crowdfunding often supports creative projects in fields such as literature, art, and media. These projects rely on the support and contributions of individuals who value and appreciate the arts and creative endeavours. Second, crowdfunding campaigns often involve storytelling and community involvement, Crowdfunding platforms provide opportunities for creators to connect with their audience, build community, and share their vision and passion. Finally, research on crowdfunding explores the motivations and behaviors of supporters and contributors ( Steigenberger 2017). By studying these aspects, researchers can gain insight into the social and cultural dynamics of Islamic crowdfunding and its impact on creative industries and communities. Research on Islamic crowdfunding involves a lot of computer scientists, because crowdfunding platforms rely heavily on website platforms and web-based online platforms ( Bouncken, Komorek, and Kraus, 2015). This platform utilizes computer science technology to facilitate fundraising from a large number of individuals or organizations. In addition, computer science plays an important role in ensuring transparency and transaction tracking in crowdfunding platforms, especially when combined with blockchain technology. Overall, computer science is an integral part of the functioning, development and analysis of Islamic crowdfunding platforms. Relationship marketing orientation, entrepreneurial awareness, system quality, and service quality significantly influence entrepreneur involvement in equity crowdfunding, Islamic crowdfunding knowledge acquisition and innovation performance. Computer science and scientist are able to answer challenges in in the fields of computers, science, and technology, but have not been able to improve the person’s ethics ( Oliver and McNeil, 2021). Overall, computer science is an integral part of the functioning, development and analysis of Islamic crowdfunding platforms.

The combination of crowdfunding platforms and blockchain technology increases transparency and transaction tracking, in line with Islamic crowdfunding ethical norms. The importance of previous successful campaigns and the quality of the website, convenience of transactions, and quality of project content will influence individual empathy as well as credibility influencing the intention to donate to charity crowdfunding ( Butticè, Colombo, and Wright, 2017). The most used keywords on Scopus document the relationship between Islamic crowdfunding and Fintech can be seen here, there needs to be further research on this matter, how does Islam view Fintech, where Fintech is a new technology, besides that it needs to be discussed why there are so many studies in Malaysia Islamic crowdfunding is a large Islamic country.

We can see the topics that were most discussed during 2013 to 2022, namely: fintech, factor, finance, and innovation. Meanwhile, based on the most keyword articles in the Scopus database, the most frequently discussed topics are: crowdfunding with 72 documents, fintech with 55, blockchain with 40, and Malaysia with 26 documents. Islamic crowdfunding initiatives contribute to the growth of Islamic crowdfunding social instruments and projects, involving stakeholders such as governments, Islamic crowdfunding financial institutions, and commercial organizations. The acceptance and success of Islamic crowdfunding is influenced by factors such as social capital, religiosity, social influence, and characteristics of Islamic communities. It is used for various purposes, including affordable housing solutions, entrepreneurship development, and investment opportunities. Islamic crowdfunding also offers an alternative model for Islamic crowdfunding venture capital firms, providing a mechanism for mitigating business risks ( Gafrej and Boujelbéne, 2022). Overall, Islamic crowdfunding is used globally for a variety of purposes, addressing economics, social and charitable needs. The multidimensional model of high-growth companies in Russia-Ukranian Islamic crowdfunding consists of several interrelated dimensions, such as innovation, finance, human resources, and business strategy after the COVID-19 pandemic is very different from the previous period ( Tchinda and Dejardin, 2021).

How can fintech be a solution and support the resilience of the Islamic crowdfunding banking sector in facing the challenges faced in the context of the COVID-19 pandemic. What are the innovation efforts made by companies in Brunei Darussalam in maintaining the resilience and continuity of their business during the pandemic ( Basir, 2022). The role of Islamic finance instruments such as waqf, in providing health equipment and supporting those affected by the pandemic has also been highlighted. Islamic fintech services are influenced by perceived ease of use, perceived usefulness, and consumer innovation. Governments and society are encouraged to strengthen and expand social networks to improve crisis management and resilience ( Pitas and Ehmer, 2020). The use of ZakaTech and its potential to increase the fundraising capacity of Islamic crowdfunding social finance institutions. The emergence of Fintech advancements has extended its influence to Islamic finance and economics, offering several solutions to mitigate the economic repercussions of COVID-19. This includes leveraging the fundamental principles and investment instruments of Islamic crowdfunding, such as profit-sharing, takaful, and endowment funds ( Benamraoui, 2021). For Islamic banking users, both knowledge of and trust in FinTech services strongly influence their decision to use Islamic crowdfunding platforms ( Irimia-Diéguez *et al*., 2023).

### The most influential studies based on keywords

Based on Table 6, it can be seen that crowdfunding, fintech, blockchain, fintech, Malaysia, Islamic Finance, and Innovation occupy the 6th largest position in the Islamic crowdfunding document. Crowdfunding is the keyword with the largest number of documents, because crowdfunding campaigns are very important for the hotel industry which is affected by the COVID-19 pandemic ( Baber *et al*., 2022). Factors that influence the success of a crowdfunding campaign include the inherent quality of the project in determining the success of the campaign ( Wan Mohamad Nazarie and Williams, 2021). Islamic crowdfunding platforms utilize block-chain technology to ensure transparency and traceability of transactions. The combination of crowdfunding platforms and blockchain technology increases transparency and transaction tracking, in line with Islamic ethical norms. The following are the most frequently cited keywords during 2013-2022.

**Table 6.** The most citation of the most topic of islamic crowdfunding.

No	Author	Title	GC
1	Khan, S. N. et al.(2021)	Blockchain smart contracts: Aplications, challenges, and future trends	150
2	Stallkamp, M., & Schotter, A. P. J (2021)	Platforms without borders? The international strategies of digital platform firms	118
3	Beaulieu, T. Y, Sarker,S., & Saker, S. (2015)	A Conceptual Framework for Understanding Crowdfunding	111
4	Aalbers, M. B. (2018).	Financial geography II: Financial geographies of housing and real estate	100
5	Grover, P., Kar, A.K., & IIavarasan, P. V. (2019)	Impact of corporate social responsibility on reputation-Insights from tweets on sustainable development goals by CEOs	94
6	Bansal, S.,Garg, I., & Sharma, G.D. (2019).	Social Entrepreneurship as a Path for Social Change and Driver of Sustainable Development: A Systematic Review and Research Agenda	94
7	Olya, H. G. T., & Gavilyan, Y. (2016).	Configurational Models to Predict Residents’ Support for Tourism Development	90
8	Barbu C. M., Florea, D. L., Dabija, D. C., & Barbu, M. C. R. (2021)	Customer Experience in Fintech	88
9	Zahed Benisi, N., Aminian, M., & Javadi, B. (2020).	Blockchain-based decentralized storage networks: A survey.	88
10	Kumar, S., Sharma, D., Rao, S., Lim, W. M., & Mangla, S. K. (2022).	Past, Present, and Future of Sustainable Finance: Insights from Big Data Analytics through Machine Learning of Scholarly Research	65

Note: *GC=*Global Citation.

### Islamic values are discussed in the islamic crowdfunding article

To examine further, what Islamic values are discussed in the Scopus document, an examination of the abstract and documents is carried out more thoroughly. The results of examining these documents can be seen in the table below. From Table 7, the most discussed Islamic values are zakat, alms, waqf, and the contracts in mua’amalah. Zakat, alms, and waqf (ZISWAF) are very important concepts in Islam, because it is an obligation for Muslims to give part of their wealth to the needy. The ZISWAF concept is a central value in Islamic economics and finance which has a direct connection with crowdfunding, because they are a source of financing that can be used to support projects that comply with sharia principles. In carrying out mu’amlah actions, Muslims cannot be separated from the mu’amalah contracts or agreements in Islam that are used in Islamic crowdfunding, such as: mudarabah, musharakah, or murabahah. A good understanding of these contracts is very important because they are the basis of sharia financing and bind the parties involved in crowdfunding transactions. In recent years, Islamic crowdfunding has developed rapidly as an alternative financing that complies with sharia principles. Therefore, research in the literature review often seeks a deeper understanding of how Islamic values, such as zakat, alms, and waqf, play a role in these developments. Muslim communities interested in Islamic crowdfunding often have a special interest in ensuring that the funds they invest or donate are in accordance with Islamic principles. Therefore, an emphasis on concepts such as zakat, alms and waqf can meet their needs and interests. The review literature may look for ways in which Islamic crowdfunding can provide positive social impact through these concepts.

**Table 7.** Islamic values in islamic crowdfunding.

No	Year	Documents	Books & Articles With Islamic Crowdfunding	Islamic value
1	2013	1	0	None
2	2014	3	0	None
3	2015	5	1	Islamic finance
4	2016	8	3	Islamic crowdfunding; lslamic Banking Service; Sharia Compliant; Islamic finance.
5	2017	23	7	Zakat, Waqf, and Sadaqah; Waqf; ljara; Supporting Social Action through Mosque; a shari’ah Compliant; Islamic crowdfunding Microfinance.
6	2018	40	22	Islamic finance; waqf crowdfunding; Islamic finance; Fintech in Islamic finance; Shari’ah compliance; Islamic crowdfunding platforms; Islamic crowdfunding on digital economy; Zakat; Islamic finance and the sharing economy; Islamic crowdfunding entrepreneurship; Islamic crowdfunding home financing through musharakah mutanaqisah.
7	2019	60	18	Cash Waqf; Crowdfunding For Islamic crowdfunding Banking; Muslim Millenial’s Intention of Donating; Islamic fintech investment innovation; sharia principles; Islamic crowdfunding Online P2P Lending Platform; Islamic crowdfunding; lslamic crowdfunding financial institutions; Shari’ah compliant microfinance; Islamic crowdfunding venture capital financing; Islamic finance regulatory; Islamic Crowdfunding; Fintech and Islamic finance.
8	2020	100	21	Islamic finance; Islamic fintech; Sadaqah; Waqf land; Waqf Islamic crowdfunding; Zakat literacy and Digital Zakat Payment; Muslim Intention in Donating; Islamic crowdfunding Banking Products; Islamic finance; Islamic banks; Islamic crowdfunding Financial Technology; Islamic crowdfunding Profit and Loss Sharing Contracts on Musharakah; Waqf; Islamic crowdfunding Tourism; Waqf Institutions; Islamic crowdfunding; A Shariah-Compliant Equity; Islamic crowdfunding Endowment Funds (Waqf); Islamic values.
9	2021	160	50	Salam-muzara’ah Linked Waqf; Shariah-Compliant Crowdfunding; Religions; Waqf Donation; Islamic crowdfunding Banking and Shariah Analysis; Islamic crowdfunding Intellectual Capital and Takaful Financial Performance; Islamic Donation; Islamic crowdfunding Banking and Shariah Analysis; Islamic crowdfunding Intellectual Capital and Takaful Financial Performance; Islamic crowdfunding Financial Industry; Muslim donating; cash waqf linked sukuk in Islamic crowdfunding Laws; Waqf land development; Islamic crowdfunding cooperation; Branding salafism; Sharia Accounting Standard; Islamic crowdfunding Economics and Finance Literatures; Zakat Management Organizations; Islamic crowdfunding Social Finance; Islamic crowdfunding Boarding Schools; Fiqh on Finance; Digital Zakat Payments; Islamic crowdfunding Economics; Equity-Based Islamic crowdfunding; Potential Failure of Islamic crowdfunding Peer-to-Peer Lending; Online lnfaq Intention; Digital Zakat Technology; Islamic crowdfunding Marketing Research; The Field of Jihadism; Islamic crowdfunding Model; Islamic fintech; Islamic fintech Consideration for Maqasid Principles; Global Zakat Cooperation Chain; Baitul Maal wa Tamwil as Integrated Islamic crowdfunding Microfinance Institution; Religious Liberty; Religiosity; Islamic crowdfunding Microfinance; Cash Waqf Fund Collection; Halal Industry’s; Halal-Friendly Sustainable Port.
10	2022	304	79	Islamic finance; Fintech in Islamic banks; Fintech and Islamic crowdfunding Financial Institutions; Fintech, Islamic crowdfunding Financial System, and Shariah Complience; Islamic crowdfunding Banking; Predlslamic crowdfundingtors of Waqf Endowment; Cash Waqf-linked SUKUK; Muslim Intention; Giving of Cash Waqf; Land Waqf, Islamic crowdfunding Financial Market and Institutions; Sharia Crowdfunding; Qard Hasan; Islamic crowdfunding Digital Economy; Muzakki and Mustahik’s collaboration model; Zakat Distribution; Waqf Management; Indonesian Muslim students; Generation Z perceptions in paying Zakat, lnfaq, and Sadaqah using Fintech; A Conceptual Development of Al-Adl Financing Risk Management; Crowdfunding and Islamic crowdfunding Securities; Foreign Muslim Workers’; Islamic crowdfunding law; Islamic crowdfunding P2P Lending; Muslim Clothing Online Purchases; Islamic crowdfunding investment decision; Sharia Financing; Online Purchases; Islamic crowdfunding investment decision; Sharia Financing; Zakat, lnfaq, and Sodaqoh (ZIS) digital payments; Islamic crowdfunding micro financial institutions; Islamic crowdfunding Social Finance; Waqf Institutions; Cash.

Islamic crowdfunding has become an increasingly important alternative in supporting projects and ventures that comply with sharia principles that involve interdisciplinary scholarship. This was triggered by the increasing demand for financing that is in accordance with Islamic values, so clear regulations are needed in Islamic crowdfunding. Good regulation can help ensure that crowdfunding platforms comply with sharia principles and protect investors and owners of Islamic Crowdfunding projects. Islamic crowdfunding can help society and the economy, contributing to poverty alleviation and the development of certain sectors through products, such as crowdfunding for small and medium enterprises, and social projects, which comply with sharia principles as the industry adapts to the diverse needs of Muslim communities. However, there are challenges in the future development of Islamic crowdfunding, namely that apart from the benefits due to increased community participation in projects that are in accordance with religious values, it has the potential risk of losing investment and compliance with sharia principles. Provide a better understanding of Islamic crowdfunding, provide valuable information for practitioners, and encourage further research in this area to support the development of better crowdfunding models in accordance with sharia principles, in order to increase the success of sharia crowdfunding in the wider community. The data is only taken from the Scopus database, and does not include the latest research that could have a significant impact on the understanding of Islamic crowdfunding, because the data is only taken up to 2022. There is a possibility of bias in literature selection which results in an incomplete or distorted representation of the relevant literature. Additionally, research on Islamic crowdfunding may vary by geographic region, so the literature review may suffer from regional bias. Many research are in English or Indonesian, but there is many research in foreign language too that became obstruction to my research.

Islamic crowdfunding is influenced by changes in the economic and legal environment, such as changes in regulations regarding sharia financing which may not be accommodated by research on Islamic crowdfunding, so this literature review also does not accommodate these changes. Further and in-depth discussion of the steps is needed. Methods are also needed to overcome the current scarcity of sharia fintech so that in the future it can be accepted by all levels of society, and increase Muslims’ understanding of openness and conformity with Islamic religious rules, especially a discipline that encompasses a profound understanding of Islamic principles and their application in various aspects of life ( al-Zuhayli, 1986).

Fiqh law is a science that discusses the detailed laws of Islamic law in the field of practical actions derived from religious evidence ., it is important to have scholars and religious scholars who have a deep understanding of social fiqh and its implementation in Islamic law. Based on the potential and limitations of sharia crowdfunding research in the data above, several recommendations are given for future sharia crowdfunding research so that it can support sharia crowdfunding, namely: the need for further and in-depth discussion regarding the steps. is needed to overcome the current shortcomings of sharia fintech so that in the future it can be accepted by all levels of society, increasing Muslims’ understanding of openness and conformity with Islamic religious rules, especially fiqh law. In this context, it is important to involve Islamic crowdfunding scholars and ulama who have a deep understanding of social fiqh and its implementation in Islamic crowdfunding law. It is also important to strengthen Islamic business ethics in Islamic crowdfunding operations, by prioritizing Islamic principles such as honesty, responsibility and maintaining consumer trust. By implementing these solutions, it is hoped that Muslims’ trust in Islamic crowdfunding can increase. To increase publication trust in the performance of sharia crowdfunding, state legislation is very necessary, so that rights, obligations, transparency and security in sharia crowdfunding are clearer.

## Conclusions

Islamic crowdfunding has become an increasingly important alternative in supporting projects and ventures that comply with sharia principles that involve interdisciplinary scholarship. This was triggered by the increasing demand for financing that is in accordance with Islamic values, so clear regulations are needed in Islamic crowdfunding. Good regulation can help ensure that crowdfunding platforms comply with sharia principles and protect investors and owners of Islamic Crowdfunding projects. Islamic crowdfunding can help society and the economy, contributing to poverty alleviation and the development of certain sectors through products, such as crowdfunding for small and medium enterprises, and social projects, which comply with sharia principles as the industry adapts to the diverse needs of Muslim communities. However, there are challenges in the future development of Islamic crowdfunding, namely that apart from the benefits due to increased community participation in projects that are in accordance with religious values, it has the potential risk of losing investment and compliance with sharia principles. Provide a better understanding of Islamic crowdfunding, provide valuable information for practitioners, and encourage further research in this area to support the development of better crowdfunding models in accordance with sharia principles, in order to increase the success of sharia crowdfunding in the wider community. The data is only taken from the Scopus database, and does not include the latest research that could have a significant impact on the understanding of Islamic crowdfunding, because the data is only taken up to 2022. There is a possibility of bias in literature selection which results in an incomplete or distorted representation of the relevant literature. Additionally, research on Islamic crowdfunding may vary by geographic region, so the literature review may suffer from regional bias. Islamic crowdfunding is influenced by changes in the economic and legal environment, such as changes in regulations regarding sharia financing which may not be accommodated by research on Islamic crowdfunding, so this literature review also does not accommodate these changes.

Further and in-depth discussion of the steps is needed. Methods are also needed to overcome the current scarcity of sharia fintech so that in the future it can be accepted by all levels of society, and increase Muslims’ understanding of openness and conformity with Islamic religious rules, especially fiqh law. In this context, it is important to involve scholars and religious scholars who have a deep understanding of social fiqh and its implementation in Islamic law. Based on the potential and limitations of sharia crowdfunding research in the data above, several recommendations are given for future sharia crowdfunding research so that it can support sharia crowdfunding, namely: the need for further and in-depth discussion regarding the steps. is needed to overcome the current shortcomings of sharia fintech so that in the future it can be accepted by all levels of society, increasing Muslims’ understanding of openness and conformity with Islamic religious rules, especially fiqh law. In this context, it is important to involve Islamic crowdfunding scholars and ulama who have a deep understanding of social fiqh and its implementation in Islamic crowdfunding law. It is also important to strengthen Islamic business ethics in Islamic crowdfunding operations, by prioritizing Islamic principles such as honesty, responsibility and maintaining consumer trust. By implementing these solutions, it is hoped that Muslims’ trust in Islamic crowdfunding can increase. To increase publication trust in the performance of sharia crowdfunding, state legislation is very necessary, so that rights, obligations, transparency and security in sharia crowdfunding are clearer.

## Data Availability

The data for this article consists of bibliographic references, which are included in the References section. Figshare: PRISMA flow diagram and checklist for ‘Bibliometric analysis of Islamic crowdfunding: a review of its journey’. https://doi.org/10.6084/m9.figshare.25563351.v2 ( Purwatiningsih *et al*., 2024). Data are available under the terms of the Creative Commons Zero “No rights reserved” data waiver (CC0 1.0 Public domain dedication).
