# Supplementary material for: Bibliometric Analysis of Islamic Crowdfunding: A Literature Review of Its Journey
Source: F1000Res. 2024 Sep 2;13:531. Originally published 2024 May 24. [Version 2] doi: 10.12688/f1000research.146797.2 (PMC11736559; doi:10.12688/f1000research.146797.2)
Supplement: Peer review history

# Reviewer report: Reviewer response for version 2

**DOI:** 10.5256/f1000research.169995.r347299

**Published:** 2024-12-23

**Authors:** Muhammad Iqmal Hisham Kamaruddin

**Affiliations:** 1 Universiti Sains Islam Malaysia, Nilai, Negeri Sembilan, Malaysia

Several comments and suggestions for improvement as follow:

 1. The sentence "Islamic crowdfunding is a relatively new business concept, even in Muslim-majority countries, and there is still limited literature on Islamic fintech", it is supposed to refer the limitation toward Islamic crowdfunding instead of Islamic fintech (para 3, page 3).

 2. Please provide full acronym name for the first introduction (e.g.: ZISWAF, para 5, page 4).

 3. It is not suitable to start the discussion under the section “crowdfunding and Islamic finance” with only highlighting the crucial of Islamic crowdfunding in hotel industry. It supposed to discuss in general before specific context will be discussed later on (para 3, page 5).

 4. The discussion under the section “crowdfunding and Islamic finance” do not interrelate between crowdfunding and Islamic finance itself. The discussion is more on crowdfunding matters only. Please elaborate more on the relationship between crowdfunding and Islamic finance.

 5. The discussion in literature review looks confusing. It is suggested to start the discussion with the background of Islamic crowdfunding. Then, continue with the relationship between Islamic crowdfunding and Islamic finance and finally the relationship between Islamic crowdfunding and fintech/blockchain.

 6. The discussion on bibliometric method used for the study is too simple. Please provide all necessary steps taken including inclusion and exclusion criteria in identifying targeted articles. Please also justify why the findings were derived from document type, source type, languages, subject area and keywords. Why others indicators such as geographical/countries are not being used?

 7. For Table 5, the sum of total publication listed is 265. However, the table stated the sum of total publication as 704. Please revise accordingly.

 8. The discussion made to elaborate findings for Table 4 and Table 5 should be in paragraphs rather than in numbering. Please revise accordingly.

 9. Under subsection “Identify the most influential studies”, it first mentioned the six most common fields of study. However, the list and the discussion only made for four most common field of study. Please revise accordingly.

 10. There are redundancy discussions and paragraphs (para 4-5, page 11 vs para 2-3, page 12). It is suggested to combine these two subsections into a single subsection.

Are the rationale for, and objectives of, the Systematic Review clearly stated?

Partly

Is the statistical analysis and its interpretation appropriate?

Not applicable

If this is a Living Systematic Review, is the ‘living’ method appropriate and is the search schedule clearly defined and justified? (‘Living Systematic Review’ or a variation of this term should be included in the title.)

Not applicable

Are sufficient details of the methods and analysis provided to allow replication by others?

No

Are the conclusions drawn adequately supported by the results presented in the review?

Partly

Reviewer Expertise:

Islamic accounting, Islamic social finance, Shariah audit, Shariah governance.

I confirm that I have read this submission and believe that I have an appropriate level of expertise to confirm that it is of an acceptable scientific standard, however I have significant reservations, as outlined above.

# Reviewer report: Reviewer response for version 2

**DOI:** 10.5256/f1000research.169995.r319839

**Published:** 2024-09-04

**Authors:** Rizka Estisia Pratiwi

**Affiliations:** 1 Universitas Islam Bandung, Bandung, West Java, Indonesia

The revised version have significantly enhanced the paper's clarity and readability. The manuscript now presents a more cohesive and persuasive argument, supported by relevant evidence and examples. The authors have successfully addressed the concerns raised in the initial review, resulting in a manuscript that meets the high standards of the journal.

Are the rationale for, and objectives of, the Systematic Review clearly stated?

Yes

Is the statistical analysis and its interpretation appropriate?

Partly

If this is a Living Systematic Review, is the ‘living’ method appropriate and is the search schedule clearly defined and justified? (‘Living Systematic Review’ or a variation of this term should be included in the title.)

Not applicable

Are sufficient details of the methods and analysis provided to allow replication by others?

Partly

Are the conclusions drawn adequately supported by the results presented in the review?

Partly

Reviewer Expertise:

Sustainable financel, islamic finance

I confirm that I have read this submission and believe that I have an appropriate level of expertise to confirm that it is of an acceptable scientific standard.

# Reviewer report: Reviewer response for version 1

**DOI:** 10.5256/f1000research.160916.r286593

**Published:** 2024-07-29

**Authors:** Muhammad Nazmul Hoque

**Affiliations:** 1 Universiti Teknologi MARA, Shah Alam, Selangor, Malaysia

The article provides a valuable contribution to understanding the landscape of Islamic crowdfunding research. However, please consider some of my advices for Improvement of this article below:

 Please provide a more detailed methodology section, including specific inclusion and exclusion criteria, data extraction methods, and detailed steps of the bibliometric analysis. This would enable replication and increase the transparency of the review process.

 Please try to find advanced statistical techniques and visualizations, such as trend analysis over time, co-authorship networks, and regression analysis to understand the factors influencing citation rates. This would enhance the interpretation of the bibliometric data.

 Please try to discuss the limitations of the study more explicitly. For instance, the reliance on Scopus as the sole database may exclude relevant studies indexed elsewhere. Acknowledging these limitations would provide a more balanced view.

 Thank you

Are the rationale for, and objectives of, the Systematic Review clearly stated?

Yes

Is the statistical analysis and its interpretation appropriate?

Partly

If this is a Living Systematic Review, is the ‘living’ method appropriate and is the search schedule clearly defined and justified? (‘Living Systematic Review’ or a variation of this term should be included in the title.)

Not applicable

Are sufficient details of the methods and analysis provided to allow replication by others?

Partly

Are the conclusions drawn adequately supported by the results presented in the review?

Yes

Reviewer Expertise:

social finance

I confirm that I have read this submission and believe that I have an appropriate level of expertise to confirm that it is of an acceptable scientific standard, however I have significant reservations, as outlined above.

# Response

**Published:** 2024-07-30

**Authors:** aris purwatiningsih

**Affiliations:** Economic Management, Universitas Dian Nuswantoro, Semarang, Central Java, Indonesia

Hi Muhammad Nazmul Hoque

 This is my answer about your questions on the review

**Inclusion and Exclusion Criteria**

**Inclusion Criteria:**

1. Publications available in the Scopus database.
2. Types of publications included: articles, book chapters, conference papers, books, reviews, editorials, short surveys, notes, and conference reviews.
3. Publications in English.
4. Publications focused on Islamic crowdfunding.
5. Publications published between 2013 and 2024.

**Exclusion Criteria:** The authors did not exclude any data to obtain a comprehensive overview of Islamic crowdfunding without excluding any type of publication. The goal is to understand all aspects of research conducted in the field of Islamic crowdfunding. Additionally, to explore all works related to Islamic crowdfunding without prejudice or strict limitations, the researchers chose not to exclude any data to gain a broader and deeper understanding of the topic. Not excluding data can reduce selection bias, where certain articles might be subjectively excluded by the researchers. By analyzing all the articles found, the results can be more objective. To reduce bias, the authors used five Research Questions (RQs). By not excluding data, the research process becomes more transparent and easier to replicate by other researchers, as all identified articles in the initial search are analyzed, making the research process and results easier to replicate and verify.

**Bibliometric Analysis**

1. Document Profile Analysis: Analyzing the profile of each relevant document.
2. Source Type Analysis: Categorizing and analyzing the types of sources from each publication.
3. Language Analysis: Analyzing the language used in the publications.
4. Subject Area Analysis: Analyzing the subject areas covered by publications related to Islamic crowdfunding.
5. Keyword Analysis: Identifying and analyzing frequently appearing keywords.
6. Publication and Citation Performance Trends Analysis: Analyzing publication trends and citation performance annually.
7. Identifying the Most Influential Studies: Identifying the most influential studies based on citations and keywords.
8. Most Common Subject Areas in Islamic Crowdfunding (2013-2024): Determining whether the three most common subjects are always present in each study over this period.
9. Islamic Values in Islamic Crowdfunding Articles: Analyzing how Islamic values are discussed in articles about Islamic crowdfunding.

**How to Determine Metrics?**

1. Identify research objectives.
2. Formulate research questions to minimize bias.
3. Establish inclusion criteria.
4. Retrieve data from Scopus.
5. Examine and categorize data.
6. Perform bibliometric data analysis.
7. Visualize data with graphs and diagrams.
8. Analyze the data.

**Detailed Bibliometric Procedures**

1. Document Profile Analysis: Analyzing the profile of each relevant document.
2. Source Type Analysis: Categorizing and analyzing the types of sources from each publication.
3. Language Analysis: Analyzing the language used in the publications.
4. Subject Area Analysis: Analyzing the subject areas covered by publications related to Islamic crowdfunding.
5. Keyword Analysis: Identifying and analyzing frequently appearing keywords.
6. Publication and Citation Performance Trends Analysis: Analyzing publication trends and citation performance annually.
7. Identifying the Most Influential Studies: Identifying the most influential studies based on citations and keywords.
8. Most Common Subject Areas in Islamic Crowdfunding (2013-2024): Determining whether the three most common subjects are always present in each study over this period.
9. Islamic Values in Islamic Crowdfunding Articles: Analyzing how Islamic values are discussed in articles about Islamic crowdfunding.

**Why use the Scopus database?**

 The Scopus database is used as a data source because it has a broad scope that includes relevant publications from various disciplines such as economics, finance, Islamic studies, and information technology. This allows for a more holistic and comprehensive analysis of how Islamic crowdfunding is studied and practiced worldwide. Additionally, Scopus provides access to reliable, comprehensive, and relevant data, which is crucial for producing a high-quality bibliometric article review.

 Thank you very much, Sir

# Reviewer report: Reviewer response for version 1

**DOI:** 10.5256/f1000research.160916.r291782

**Published:** 2024-06-29

**Authors:** Rizka Estisia Pratiwi

**Affiliations:** 1 Universitas Islam Bandung, Bandung, West Java, Indonesia

**Are sufficient details of the methods and analysis provided to allow replication by others?**

 The methods section lacks the detail necessary for replication, the authors do not specify:

- The search terms used beyond the keyword "Islamic crowdfunding."
- The inclusion and exclusion criteria for selecting articles.
- The specific databases or sources used beyond Scopus.
- The methods used to extract and analyze data from the articles.

**Is the statistical analysis and its interpretation appropriate?**

 This article does not present any statistical analysis. It is a literature review that uses descriptive statistics (e.g., counts and percentages) to summarize the characteristics of the literature on Islamic crowdfunding (e.g., publication year, source type, language). However, it does not employ inferential statistics to test hypotheses or make generalizations beyond the included studies.

**Are the conclusions drawn adequately supported by the results presented in the review?**

*Partly. *There is some unsupported, or even overreaching conclusions:

- Impact on poverty alleviation and economic development (the conclusion that Islamic crowdfunding contributes to poverty alleviation and economic development is not directly supported by the results presented in the review. While the authors mention these potential benefits, the review does not provide concrete evidence or data to substantiate these claims)
- Need for specific Fiqh regulatory guidelines and government support (the conclusion calling for specific Fiqh regulatory guidelines and government support, while potentially valid, is not explicitly derived from the results of the literature review. It appears to be more of a recommendation or suggestion based on the authors' interpretation of the challenges identified in the literature)

Some claims need more direct evidence from the reviewed literature. The authors could strengthen their conclusions by providing more specific examples and data from the studies they reviewed to support their assertions about the impact of Islamic crowdfunding and the need for regulatory and government support.

**If this is a Living Systematic Review, is the method appropriate and is the search schedule clearly defined and justified? ('Living Systematic Review' or a variation of this term should be included in the title.)**

 Obviously, this article is not a LSR, because the authors do not provide the updated data and recent studies.

Are the rationale for, and objectives of, the Systematic Review clearly stated?

Yes

Is the statistical analysis and its interpretation appropriate?

Partly

If this is a Living Systematic Review, is the ‘living’ method appropriate and is the search schedule clearly defined and justified? (‘Living Systematic Review’ or a variation of this term should be included in the title.)

Not applicable

Are sufficient details of the methods and analysis provided to allow replication by others?

Partly

Are the conclusions drawn adequately supported by the results presented in the review?

Partly

Reviewer Expertise:

Sustainable financel, islamic finance

I confirm that I have read this submission and believe that I have an appropriate level of expertise to confirm that it is of an acceptable scientific standard, however I have significant reservations, as outlined above.

# Response

**Published:** 2024-07-30

**Authors:** aris purwatiningsih

**Affiliations:** Economic Management, Universitas Dian Nuswantoro, Semarang, Central Java, Indonesia

Hi Rizka,

 This is my answer for your questions on my article:

  

- The search terms used beyond the keyword "Islamic crowdfunding."

 

- **Search terms** islamic AND crowdfunding AND PUBYEAR > 2012 AND PUBYEAR < 2023

 

- Inclusion and exclusion criteria for selecting articles.

**Inclusion and Exclusion Criteria**

**Inclusion Criteria:**

1. Publications available in the Scopus database.
2. Types of publications included: articles, book chapters, conference papers, books, reviews, editorials, short surveys, notes, and conference reviews.
3. Publications in English.
4. Publications focused on Islamic crowdfunding.
5. Publications published between 2013 and 2024.

**Exclusion Criteria:** The authors did not exclude any data to obtain a comprehensive overview of Islamic crowdfunding without excluding any type of publication. The goal is to understand all aspects of research conducted in the field of Islamic crowdfunding. Additionally, to explore all works related to Islamic crowdfunding without prejudice or strict limitations, the researchers chose not to exclude any data to gain a broader and deeper understanding of the topic. Not excluding data can reduce selection bias, where certain articles might be subjectively excluded by the researchers. By analyzing all the articles found, the results can be more objective. To reduce bias, the authors used five Research Questions (RQs). By not excluding data, the research process becomes more transparent and easier to replicate by other researchers, as all identified articles in the initial search are analyzed, making the research process and results easier to replicate and verify.

  

- The specific databases or sources used beyond Scopus.

**Why use the Scopus database?**

 The Scopus database is used as a data source because it has a broad scope that includes relevant publications from various disciplines such as economics, finance, Islamic studies, and information technology. This allows for a more holistic and comprehensive analysis of how Islamic crowdfunding is studied and practiced worldwide. Additionally, Scopus provides access to reliable, comprehensive, and relevant data, which is crucial for producing a high-quality bibliometric article review.

  

- The methods used to extract and analyze data from the articles.

**Detailed Bibliometric Procedures**

1. Document Profile Analysis: Analyzing the profile of each relevant document.
2. Source Type Analysis: Categorizing and analyzing the types of sources from each publication.
3. Language Analysis: Analyzing the language used in the publications.
4. Subject Area Analysis: Analyzing the subject areas covered by publications related to Islamic crowdfunding.
5. Keyword Analysis: Identifying and analyzing frequently appearing keywords.
6. Publication and Citation Performance Trends Analysis: Analyzing publication trends and citation performance annually.
7. Identifying the Most Influential Studies: Identifying the most influential studies based on citations and keywords.
8. Most Common Subject Areas in Islamic Crowdfunding (2013-2024): Determining whether the three most common subjects are always present in each study over this period.
9. Islamic Values in Islamic Crowdfunding Articles: Analyzing how Islamic values are discussed in articles about Islamic crowdfunding.

Also for your question on Impact on poverty alleviation and economic development and Fiqh Regulatory in Indonesia this is my Answer:

**Poverty Alleviation And Economic Development Resulting From Islamic Crowdfunding**

                   Islamic crowdfunding platforms can serve as a funding solution for SMEs and startups, facilitating access to capital for businesses that may struggle to obtain traditional financing (Hendratmi et al., 2019). The concept of crowdfunding, when connected with spiritual and religious responsibilities, can drive the economic growth of micro and small enterprises, thereby contributing to economic development (Aderemi & Ishak, 2020).Islamic values ​​and principles, when applied in crowdfunding initiatives, can help households improve their livelihoods and reduce poverty by promoting ethical financial practices and responsible wealth distribution. Islamic crowdfunding is in line with the principles of Islamic finance, promoting equitable distribution of wealth and avoiding riba, which can contribute to poverty reduction. Islamic crowdfunding offers a Shariah-compliant and ethical financing solution that can empower the underprivileged, strengthen economic foundations, and promote sustainable development. Ultimately, it plays a vital role in poverty alleviation efforts in Muslim societies and beyond. By leveraging the concepts of cooperation, risk sharing, and ethical investment, Islamic crowdfunding can contribute to poverty alleviation in a variety of ways (Aderemi & Ishak, 2022). Islamic crowdfunding platforms, such as Qard Hasan crowdfunding, can create opportunities for micro-enterprises and bridge the wealth gap between different socio-economic classes, encouraging collaboration and a more charitable society (Aderemi & Ishak, 2022). By providing funding for small and medium enterprises (SMEs) and social projects tailored to meet the needs of Muslims, Islamic crowdfunding contributes to poverty alleviation and sectoral development. In addition, Islamic crowdfunding can reduce the government's financial burden in supporting micro-enterprises, empowering them to be more independent, creative, and competitive in promoting their products (Ishak, 2023).

                   Indonesia, as the country with the largest Muslim population in the world, has developed policies and regulations to support the growth of Islamic crowdfunding, involving the Financial Services Authority (OJK) and the National Sharia Finance Committee (KNKS). The OJK regulates and supervises the financial industry, including Islamic crowdfunding, through regulations such as OJK Regulation No. 37/POJK.04/2018 concerning equity crowdfunding services based on information technology, which includes Sharia-compliant platforms. KNKS, now known as the National Committee for Islamic Economy and Finance (KNEKS), plays a role in developing and promoting policies and regulations to strengthen the Sharia economy and finance, including crowdfunding, with support from the Indonesian government through various Sharia fintech initiatives. The National Sharia Council (DSN-MUI) also plays a crucial role by issuing fatwas, such as Fatwa No. 117/DSN-MUI/II/2018 and No. 118/DSN-MUI/II/2018, to ensure that crowdfunding products and services comply with Sharia principles, and each Islamic crowdfunding platform in Indonesia must have a Sharia Supervisory Board to ensure adherence to Sharia principles in their operations and offered products.

 According to OJK, which oversees Indonesia's financial sector, several Sharia-compliant crowdfunding platforms, such as Ethis Indonesia, ALAMI, and SyarQ, are registered with and supervised by the authority. Ethis Indonesia focuses on property and social financing, ALAMI provides Sharia-compliant P2P lending for SMEs, and SyarQ offers Sharia-based consumer financing.. The transaction volume of Islamic crowdfunding in Indonesia has shown significant growth, with ALAMI recording over IDR 1 trillion in financing by the end of 2023, supporting thousands of SMEs across Indonesia. The sectors financed through Islamic crowdfunding platforms include micro, small, and medium enterprises (MSMEs), property, education, and social projects. Investor participation in Islamic crowdfunding platforms continues to increase, indicating high confidence in Sharia-compliant financing mechanisms. The use of technology in Sharia crowdfunding platforms facilitates investment and financing processes, enhances transparency, and reduces risk.

 Aderemi, A. M. R. and Ishak, M. S. I. (2020). Crowdfunding as an alternative mode of financing for micro and small enterprises: a   proposed qard-al-hasan contract. International Journal of Islamic Economics and Finance (IJIEF), 3(3).        https://doi.org/10.18196/ijief.3235.

 Aderemi, A. M. R. and Ishak, M. S. I. (2022). Qard hasan as a feasible islamic financial instrument for crowdfunding: its potential    and possible application for financing micro-enterprises in malaysia. Qualitative Research in Financial Markets, 15(1), 58-              76. https://doi.org/10.1108/qrfm-08-2021-0145.

 Fang, Y. (2021). The future path to china’s poverty reduction—dynamic decomposition analysis with the evolution of china’s          poverty reduction policies. Social Indicators Research, 158(2), 507-538. https://doi.org/10.1007/s11205-021-02716-5

 Hendratmi, A., Ryandono, M. N. H., & Sukmaningrum, P. S. (2019). Developing islamic crowdfunding website platform for startup companies in indonesia. Journal of Islamic Marketing, 11(5), 1041-1053. https://doi.org/10.1108/jima-02-2019-0022.

 Idemudia, U. (2008). Oil extraction and poverty reduction in the niger delta: a critical examination of partnership initiatives. Journal               of Business Ethics, 90(S1), 91-116. https://doi.org/10.1007/s10551-008-9916-8

 Ishak, M. S. I. and Mohammad Nasir, N. S. (2023). The applicability of islamic crowdfunding as an alternative funding for micro-     entrepreneurs in malaysia. Qualitative Research in Financial Markets, 16(3), 548-564. https://doi.org/10.1108/qrfm-12-  2022-0202

*List of Equity Crowdfunding Platforms that Have Obtained Permits from OJK.* (2019). Retrieved July 30, 2024, from           https://ojk.go.id/id/berita-dan-kegiatan/publikasi/Pages/Daftar-Platform-Equity-Crowdfunding-yang-Telah-           Mendapatkan-Izin-dari-OJK.aspx

 Liu, Y. and Xu, Y. (2016). A geographic identification of multidimensional poverty in rural china under the framework of sustainable                livelihoods analysis. Applied Geography, 73, 62-76. https://doi.org/10.1016/j.apgeog.2016.06.004.

 Liu, Y., Guo, Y., & Zhou, Y. (2018). Poverty alleviation in rural china: policy changes, future challenges and policy implications. China                Agricultural Economic Review, 10(2), 241-259. https://doi.org/10.1108/caer-10-2017-0192.

 Wang, F., Du, L., Tian, M., Liu, Y., & Zhang, Y. (2023). Sustainability of rural tourism in poverty reduction: evidence from panel data                 of 15 underdeveloped counties in anhui province, china. Plos One, 18(3), e0283048.     https://doi.org/10.1371/journal.pone.0283048

 Zhang, H., Wu, K., Qiu, Y., Chan, G., Wang, S., Zhou, D., … & Ren, X. (2020). Solar photovoltaic interventions have reduced rural      poverty in china. Nature Communications, 11(1). https://doi.org/10.1038/s41467-020-15826-4

 Thank you very much
